# Biomass and carbon estimation for scrub mangrove forests and examination of their allometric associated uncertainties

**DOI:** 10.1371/journal.pone.0230008

**Published:** 2020-03-10

**Authors:** Paulo César Costa Virgulino-Júnior, Diego Novaes Carneiro, Wilson Rocha Nascimento, Michele Ferreira Cougo, Marcus Emanuel Barroncas Fernandes

**Affiliations:** 1 Universidade Federal do Pará, Campus Bragança, Laboratório de Ecologia de Manguezal, Bragança, Pará, Brazil; 2 Instituto Tecnológico Vale, Belém, Pará, Brazil; 3 Universidade Federal do Pará, Instituto de Geociências, Cidade Universitária, Belém, Pará, Brazil; Eidgenossische Forschungsanstalt fur Wald Schnee und Landschaft Institut fur Schnee- und Lawinenforschung, SWITZERLAND

## Abstract

Reliable estimates of biomass and carbon storage are essential for the understanding of the environmental drivers and processes that regulate the productivity of scrub forests. The present study estimated total (above-ground, AGB + below-ground, BGB) biomass and carbon storage of a scrub forest dominated by *Avicennia germinans* (L.) L. based on the existing allometric models for the AGB, while novel models were developed to estimate the BGB. Data collection followed a destructive approach by using the "sampling method", from 45 trees divided into three height classes. Tree height and diameter were used to estimate the BGB of these forests, providing more accurate estimates of their biomass. Our findings indicate the existence of a direct relationship with increasing topography and interstitial salinity, which result in an increase in the percentage contribution of the AGB. By contrast, increasing topography also led to reduction in tree height and contribution of the BGB, although this compartment represents approximately half of the total biomass of these forests. The contribution of BGB estimates increased from 43 to 49.5% from the lowest to the highest height class and the BGB and AGB values reached approximately 87 Mg ha^-1^ (48.6%) and 91.7 Mg ha^-1^ (51.4%), respectively. The estimates of the biomass and carbon stocks of scrub mangroves vary considerably worldwide, which reflects the uncertainties derived from the application of distinct sampling methods. Specific models developed for each height class should be considered instead generalist models to reduce the general uncertainties on the production and distribution of biomass and the storage of carbon. Overall, our results overcome a major lacuna in the development of allometric equations to estimate the production of BGB and the storage of carbon by scrub mangrove forests, contributing to the refinement of the total biomass estimates for this type of mangrove forest.

## Introduction

Salinity and water deficit are the principal environmental drivers of stress in mangrove tree species [1,2]. Despite these limiting factors, mangrove forests are able to form successfully through the adoption of unique ecological strategies by the tree species that make up this system, with the tolerance of specific conditions being determined by the optimal range within an entire gradient of conditions [3–5]. In mangrove systems, trees of the genus *Avicennia* L. are considered to be the most tolerant of salinity [6], although the potential of these trees for growth and the assimilation of carbon is reduced with increasing salinity [7]. In comparison with the other Neotropical mangrove species, *Avicenna germinans* (L.) L. is one of the most resilient forms, capable of tolerating an extensive gradient of salinity [8,9]. The species has achieved this through the development of a number of morphological and ecophysiological adaptations [3,10,11]. The stress generated by conditions of extreme salinity affects the structure of the mangrove, reducing its stature, trunk diameter, and the size of the leaves [12,13], transforming the forest into a scrub mangrove, which is unlike the dwarf mangrove, where reduced stature is not accompanied by a reduction in leaf size, for example [14].

The mangroves on the Brazilian Amazon coast occupy a number of distinct gradients of soil salinity and topography [15]. On the Ajuruteua Peninsula, in the state of Pará, for example, the patches of scrub mangrove forest are dominated almost entirely by *A*. *germinans*, which occupies the sites with the highest salinity and topography, forming a gradient from shrub-like trees to short mangrove trees [16]. These environmental drivers also have a direct influence on the production of biomass and carbon storage [17], with the trees distributing their nutritional resources as efficiently as possible in response to these conditions [18]. Studies of biomass production and carbon storage have focused on different types of mangrove forest around the world, including fringe [19], basin [20], riverine [21], and scrub [19] forest types, which have generated an ample range of estimates (~ 8–460 Mg ha^-1^), reflecting the diversity of environmental conditions. Most estimates of the production of biomass and carbon storage by mangrove ecosystems have focused on well-developed forests [22–26], while the effects of stressful conditions have been largely overlooked. As few studies have focused specifically on these stressed forests of short stature [19,27,28], it is important to develop allometric equations that provide reliable estimates of their biomass, not only because this vegetation is characterized by considerable morphological variation [29] and is widely distributed in tropical and subtropical regions [30], but also because these equations will help to minimize the uncertainties intrinsic to the estimates of productivity available for the world’s mangrove ecosystems as a whole [31].

In general, studies of the production of biomass by mangrove forests, including stressed forests [27,28,32,33], have focused on the above-ground biomass (AGB) [21,34–36], while only a few studies have analyzed the below-ground biomass (BGB) [37,38], and none have focused specifically on the BGB of scrub and/or dwarf mangrove forests. Below-ground biomass is considered to be one of the five primary carbon reserves in forested areas [39], and represents one of the largest carbon stocks in the tropical region [40]. To fill this knowledge gap, we designed a study to assess the estimates of the above- and below-ground biomass and carbon storage in the different compartments and height classes of these forests dominated by *A*. *germinans* on the Ajuruteua Peninsula, on the Brazilian Amazon coast. The collection of data on the topography and salinity of these sites allowed us to identify the principal environmental drivers of the variation in the forest height classes. Similarly, the collection of data on root biomass directly through the excavation of specimens allowed us to develop allometric models to estimate the BGB of these forests and to assess the estimates of the total biomass (AGB+BGB) of scrub mangrove forests. Through this approach, we aimed to provide the means for the calculation of reliable biomass estimates that can be extrapolated to other, similar mangrove forests around the world. In addition to our estimates, derived from different height classes of scrub mangrove trees, we developed a general model comprising all height classes to assess the possibility of minimizing uncertainties associated with allometric models for biomass/carbon estimates.

## Materials and methods

### Study site

**Fig 1:**
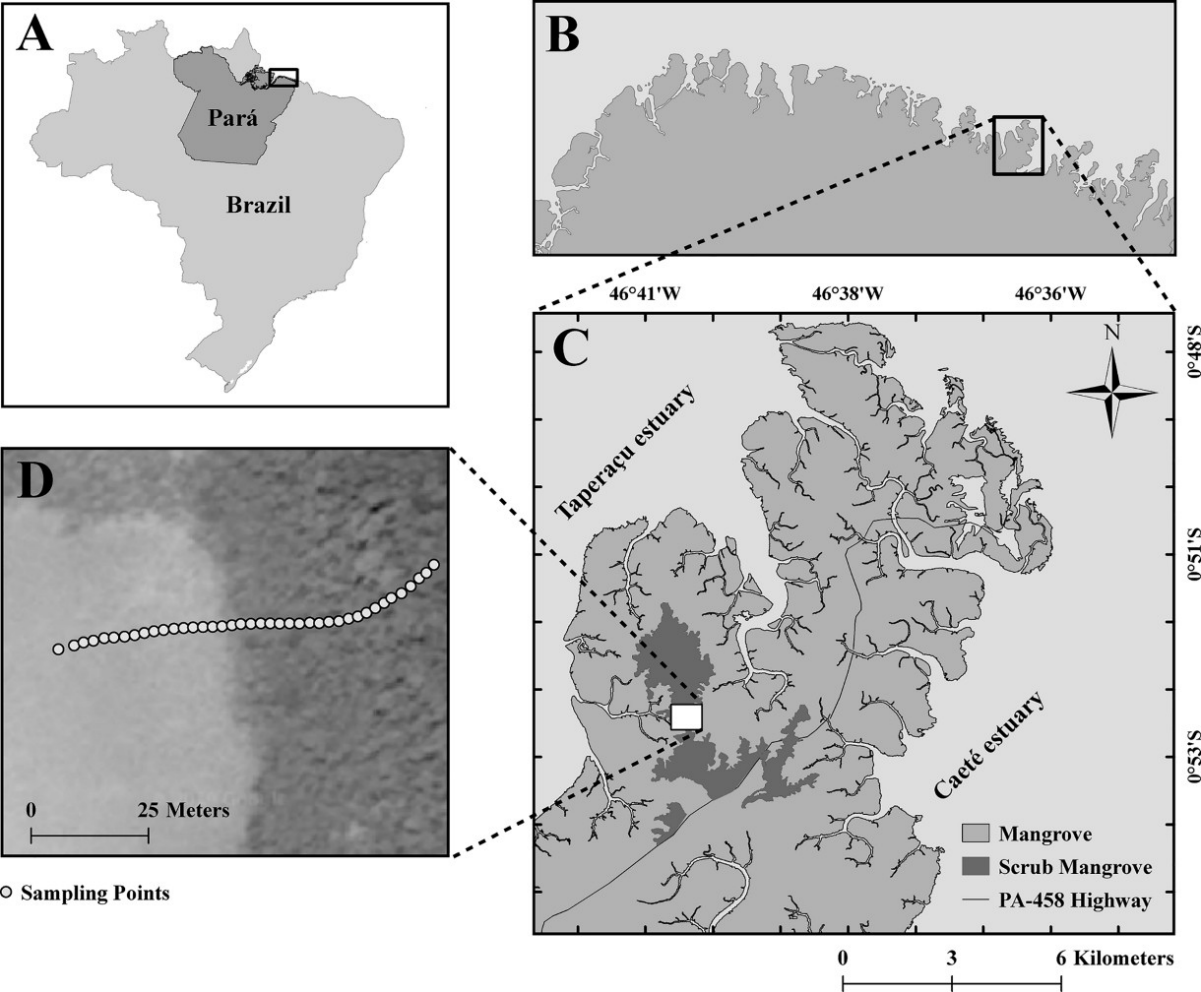
Map of the study site. a = Brazil, showing the state of Pará, with the coastal area of the state, outlined by the black rectangle b = Pará showing the coastal area of the Bragança microregion, outlined by the black rectangle, c = the Ajuruteua Peninsula, in the municipality of Bragança, showing the scrub *Avicennia germinans* forest (hatched area), and d = the sampling points for the collection of the data on salinity and topography.

The study site is located on the Ajuruteua Peninsula (00°45’–01°07’ S, 46°50’–46°30’ W), in the northeastern extreme of the state of Pará, on the Brazilian Amazon coast (Fig 1), a region dominated by a hot and humid equatorial climate. The climatic data for the past 40 years reveal a mean annual temperature of 26.5ºC, mean annual precipitation of 2,348.5 mm, and relative humidity of 85% [41]. The region has two well-defined climatic periods [42], with the timing of the rainy season being influenced primarily by the location of the Intertropical Convergence Zone, or ITCZ [43]. The rainy season occurs when the ITCZ shifts southward between January and June, whereas the dry season (monthly precipitation of less than 100 mm) lasts from July through December [44].

On this peninsula, a total area of approximately 16,465.5 ha (= 164.65 km^2^) is covered by mangrove forest, which is formed by three tree species: *Rhizophora mangle* L., *Laguncularia racemosa* (L.) C. F. Gaertn., and *A*. *germinans*. The region is characterized by semidiurnal macrotides [45], with a tidal range of 4–6 m [46]. Most input of freshwater comes from the Caeté River which, during the rainy season, reduces salinity to zero in the local tidal creeks, such as the Taici Creek, which traverse the mangroves bordering the upland forests (Fig 1). This figure also shows the central portion of the peninsula, at kilometer 21 of the PA-458 state highway, where scrub mangrove forests dominate the landscape as a result of both high topography and the low flooding frequency, which is reflected in a hydrological deficit and increased salinity (> 100).

### Soil salinity and topography

The topographic gradient was surveyed using a differential Trimble R4 GNSS handheld GPS [47] and a Topcon ES series total station [48]. The total station was used to sample 45 points (Fig 1D), with the angles and distances being measured using an electronic optical rangefinder and an electronic angle scanner [49]. The geographic coordinates were recorded using the differential GPS in static post-processed mode, with a posteriori correction by triangulation using the geodesic stations of the Brazilian Continuous Monitoring Network, or RBMC [50]. The data collected using the GNSS receptor were processed using the Trimble Business Center software [51], while the coordinates collected by the total station were corrected using the Spectrum link software [52]. The salinity of the soil was measured subsequently along the topographic gradient. For this, samples of interstitial water were extracted from a depth of 30 cm using a pipette inserted through a 200 mm diameter PVC tube, which was buried in the ground.

### Tree allometric dataset

The mangrove tree species, *A*. *germinans*, forms the scrub mangrove forests located on the highest part of the peninsula, that is, at 3.5 m a.s.l. (Fig 1), covering an area of approximately 812 ha, which represents around 5% of the total area of mangroves on the peninsula. These stressed mangrove forests present a gradient of structural features, with shrub-like trees of heights as low as 30 cm, many twisted branches resulting from regrowth, and stunted trees, with some individuals of up to 800 cm in height [16].

The structural characteristics of a forest can provide important parameters for the development of allometric equations, although the results of destructive sampling can provide more realistic values [53]. This supports the destructive sampling procedures adopted in the present study, which provide more accurate parameters for the application of the allometric equations than the data available from other sites. Thus, mangrove trees with different classes of height were cut, according to the Instituto Chico Mendes de Conservação da Biodiversidade (ICMBio), Licence Nº 60471/2017.

The scrub mangrove forest was divided in three well-defined strata, with tree heights of (i) 30–120 cm, (ii) >120–250 cm, and (iii) > 250, and emergent trees of up to 800 cm [16]. Considering the destructive approach of the sampling method, we measured 15 trees for each height class, which is a representative sample number for BGB studies [38], with the equivalence of the samples being the basis for the comparative effect between the height classes. The choice of the sampled trees was based on the variation existing in the range of each height class, that is, the height of the collected trees was well distributed within each class, reducing the sampling bias. The selected trees were well-separated from their neighbors along the scrub mangrove forest that covers an area of approximately 812 hectares, in order to facilitate the excavation of their roots. A series of measurements were taken from each tree: (i) total height (h; m); (ii) diameter at breast height (DBH), that is, 130 cm above the ground, in the case of trees that were at least 3.5 m in height [54]; (iii) basal diameter (bd), that is, at 30 cm above the ground, in the case of trees that were less than 3.5 m in height [55]; (iv) area of the tree crown, given by the formula: crown area = [(R1)/2)*(R2)/2)]*π], where R1 = the greatest radius and R2 = the smallest radius, and (v) crown volume, given by the formula: crown volume = crown area*h [28].

We obtained a disk sample of each tree at base height (30 cm above the ground), from which a transverse section was extracted to determine the density of the wood (*ρ*; g cm^-3^), based on the water displacement method [56]. The dry mass was obtained after drying the sample in an oven at 70ºC for 72 hours or until reaching a constant weight. The basic density of the wood was obtained using the equation: *ρ* = M/V, where M = the dry mass (g) and V = the volume (cm^3^).

The BGB was quantified by root sampling [38]. The below-ground portion of the *A*. *germinans* trees was divided into three compartments: (i) root crown, (ii) primary roots, which originate from the root crown, and (iii) secondary roots, which originate from the primary roots. Trees were cut at a height of 15 cm above the ground to facilitate the excavation and removal of the root crown. The primary roots were exposed from their origin at the root crown to their deepest extremity. We extracted two primary roots from each tree, including the pneumatophores, only when these were buried, and their associated secondary roots. The roots were then taken to the Mangrove Ecology Laboratory on the Bragança campus of the Federal University of Pará, where the basal diameter and length of the primary and secondary roots were measured. The samples were then washed throughly and carefully, and their fresh weight was determined using a digital precision balance (0.02 kg). This material was divided into subsamples that were weighed to determine their fresh weight using a second digital precision balance (0.01 g). The subsamples were dried in an oven at 105ºC until they reached a constant weight, and they were then weighed again to determine their dry weight. The dry (Dry = *D*) to fresh (Fresh = *F*) weight ratio (*D*:*F*) was also calculated for every root compartment of each tree.

The biomass values obtained for the excavated primary and secondary roots were used to develop allometric models to estimate the dry weight of the portion of each type of root that was not excavated. For this, we used the basal diameter of each excavated root as the predictor variable. The total dry weight of the primary and secondary roots was determined from the sum of the dry weights recorded for the “excavated roots” and the dry weights estimated for the “unexcavated roots”.

The total dry weight of the primary and secondary roots was then added to the dry weight of the root crown to obtain the total below-ground biomass of each tree. For this, we used the structural attributes of each tree (height, trunk diameter, crown area and volume, and the density of the wood) as the predictor variables for the development of the allometric models used to estimate the total BGB for the three tree height classes of the *A*. *germinans* scrub forest. We used a similar approach to estimate the AGB for the scrub forest, using the allometric models developed previously for the same study site [16] (S1 Table). Finally, to transform the BGB values into carbon, we used the carbon concentration (42.6%) recorded for *Avicennia schaueriana* Stapf & Leechman *ex* Moldenke in the mangroves of the Brazilian Southeast [57]. The AGB was converted based on the carbon concentration (41.9%) estimated by Carneiro [16] for the same scrub *A*. *germinans* forest study area.

### Data analysis

The weight of the “unexcavated” primary and secondary roots of each height class was estimated using models developed specifically for this purpose. As the relationship between the dry weight of the primary/secondary roots and the basal diameter was non-linear, since in biomass data it is often a power function of a variable that express tree size [58], a number of different regressions were applied to describe these relationships. The power (y = a*x^b^) and second-order polynomial (y = ax^2^+bx+c+e) functions were the best models, where y = the dry weight of the primary or secondary root (kg root^-1^), x = the basal diameter of the root (cm), a, b and c = the model parameters, and e = the additive error of the model.

The normality of the residuals of each regression was verified using the Shapiro-Wilk test and the homoscedasticity was verified using the Breusch-Pagan test and the graphic analysis of the residuals. We selected the best model to estimate the biomass of the “unexcavated” primary and secondary roots based on the goodness of fit tests: (i) adjusted coefficient of determination (R^2^_adj_), (ii) Root Mean Square Error (RMSE) and (iii) Mean Percentage Error (MPE). Afterwards, we developed linear and non-linear allometric equations to estimate the BGB and calculated R^2^_adj_, RMSE, MPE, and Akaike’s Information Criterion (AIC) to define the best statistical model for each tree height class:

- R^2^_adj_
  $$R_{adj}^{2}=1-\left[\frac{\left(1-R^{2})*(n-1\right)}{n-k-1}\right]$$ (1)
- Root Mean Square Error (RMSE):
  $$RMSE=\sqrt{\frac{\sum e^{2}}{n}}$$ (2)
- Mean Percentage Error (MPE).
  $$MPE=\left(\frac{\sum (e)/n}{M_{obs}}\right)*100$$ (3)
- Akaike’s Information Criterion (AIC):
  $$AIC=n*\left(ln(\frac{\sum \left(e^{2}\right)}{n})\right)+2*(k+1)+c$$ (4)
  where: *n* is the number of samples, *k* is the number of independent variables present in the model, *R*^*2*^ is the coefficient of determination, the term “*e*” refers to the residuals, that is, the difference between the observed and predicted values, *M*_*obs*_ is the average observed dry weight, and *c* is the constant.

The total BGB values estimated for each height class were compared using the nonparametric Kruskal-Wallis analysis of variance (H), with Dunn’s *post hoc* test. This same procedure was used to compare the contribution of each compartment to the BGB among the different height classes. The variation in the BGB values between different compartments [root crown and roots (primary+secondary)] in each height class was verified using the t test. All the analyses were run in the R studio 3.6.0 platform [59]. The non-linear allometric equations were developed using the *nls2* package [60] and the *post hoc* test in *FSA* package [61], both in the R platform.

## Results

### Soil salinity and topography

**Fig 2:**
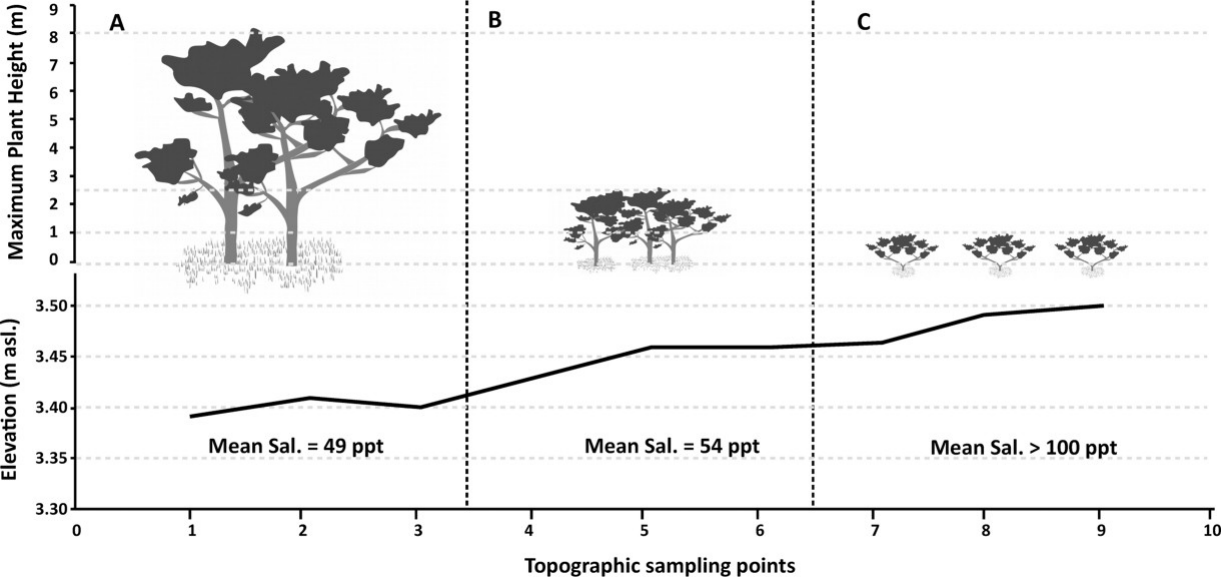
Relationship between environmental drivers (topography and salinity) and tree height across the distribution of the *Avicennia germinans* scrub forests on the Ajuruteua Peninsula in Bragança, Brazil. The trees and shrubs are represented by their respective classes (A = Class 3, B = Class 2, C = Class 1). The hand held refractometer (RHS-10/ATC) used in this assessment has a measuring range of up to 100% ppt.

The scrub mangrove forest varied considerably in height across its distribution on the Ajuruteua Peninsula as a result of the variation in both topography and the salinity gradient (Fig 2). Our results revealed that topography-driven salinity reduces tree height by approximately 7 meters, and changes the habit (i.e., shape and growth) [62] of the *A*. *germinans* individuals, with individuals in height class 1 presenting a bushy habit, with multiple stems. The reduction in height was inversely related to both the increasing topographic gradient (elevation increasing 0.13 m, from 3.39 m to 3.52 m a.s.l.) and interstitial salinity, which increased 55 ppt, from 45 to 100 ppt, the maximum reading of the RHS-10/ATC refractometer (Fig 2).

### Belowground biomass allometry

The three height classes of the scrub mangrove presented different patterns of BGB according to the models developed for the prediction of the dry weight of the unexcavated primary and secondary roots, with all parameters estimated being significantly different at the 1% level. Similarly, the values of all the selection criteria of the models developed using the residuals for validation, indicated that the equations selected have high predictive power. In general, the models generated for height class 3 were the most accurate in comparison with the other two classes (Table 1). The models developed for the primary and secondary roots of this class were the best adjusted (R^2^_adj_ = 0.98). However, when the two models are compared, the lowest RMSE value (0.005) was recorded for the secondary root model. The coefficients of determination (R^2^_adj_) explained between 86% and 98% of the variance in the biomass observed in each height class analyzed and in each type of root. All the models presented low MPE values (-0.75–0.23%), where the negative values indicate underestimates, and the positive values, overestimates (S2 Table). The residuals of all the models were normally distributed and had homogeneous variances.

**Table 1:** The allometric equations used to estimate the dry weight (kg) of the unexcavated roots of the scrub *Avicennia germinans* trees in the three height classes.

Class	Compartment	n	Coefficient			RMSE	R^2^_adj_	MPE
			a	b	c			
C1	Secondary root *	17	0,0236337	-0,0052021	0,0001993	0,002	0,94	-0,8
C1	Primary root	13	0,0272117	2,5584838	-	0,006	0,86	-0,12
C2	Secondary root	32	0,0456687	3,4475717	-	0,004	0,97	-0,75
C2	Primary root	22	0,0873721	1,6762250	-	0,084	0,86	-0,17
C3	Secondary root	9	0,0413852	3,7995342	-	0,005	0,98	0,20
C3	Primary root	22	0,0075510	3,1628440	-	0,348	0,98	0,23

The model used was: y = a*x^b^

*The model used was: y = a*x^2^ + b*x+c

n = the number of samples, RMSE = Root Mean Square Error, R^2^_adj_ = the adjusted coefficient of determination, MPE = Mean Percentage Error.

As for the models used to estimate the unexcavated roots, the coefficients of all the models selected to estimate the total BGB and that of the different compartments were significant (Table 2). These models also had a high degree of predictive power, and the residuals were also distributed normally and had homogeneous variances (S3 Table). While a number of linear and non-linear relationships were tested, the linear and power equations were the most adequate. In the case of height classes 1 and 2, the best equations were found for the crown, based on the selection criteria used (RMSE = 0.002 kg; AIC = -80.74; R^2^_adj_ = 0.98 for class 1, and RMSE = 0.03 kg; AIC = -27.50; R^2^_adj_ = 0.95 for class 2). In the case of height class 3, the best equation was that developed for the total biomass, considering the same criteria (RMSE = 0.16 kg; AIC = 4.93; R^2^_adj_ = 0.99). The MPE values ranged from -0.23% to 0.01% among the different classes, with the values related to class 3 representing underestimates, while those referring to class 1 represented overestimates.

**Table 2:** Allometric models used to estimate the total, root (primary+secondary), and root crown below-ground biomass of the three height classes.

Class	Compartment	Model	n	Coefficient					RMSE	AIC	R^2^_adj_	MPE
				a	b	c	d	e				
C1	Root	y = a+b*h*+cρ	8	0.114	0.005	-0.682			0.03	-31.84	0.93	0.008
	Root crown	y = a+b*h*+cD+dV	9	-0.021	0.000	0.021	6.53E-08		0.002	-80.74	0.98	-0.077
	Total	y = a*b*h*+cD+dρ	8	0.038	0.002	0.082	-0.3781		0.02	-31.65	0.92	0.001
C2	Root	y = a*D^b^	11	0.465	1.024				0.37	15.45	0.94	-0.033
	Root crown	y = a+b*h*+cD+dV+eρ	8	0.814	-0.003	0.017	7.27E-08	-0.660	0.03	-27.50	0.95	-0.083
	Total	y = a*D^b^	11	0.468	1.036				0.41	17.76	0.93	-0.038
C3	Root	y = a*D^b^*h^c^*V^d^*ρ^e^	8	0.003	-2.180	2.572	-0.024	5.275	0.55	25.17	0.98	-0.058
	Root crown	y = a*D^b^*h^c^*V^d^	9	8.63E-10	1.138	2.730	0.117		0.24	10.12	0.92	-0.231
	Total	y = a*D^b^*h^c^*V^d^*ρ^e^	8	0.002	-1.381	2.205	0.005	4.263	0.16	4.93	0.99	0.003

n = number of samples, D = diameter of the stem (cm), *h* = total height (cm), V = volume (cm^3^), *ρ* = wood density; a, b, c, d, e = the regression coefficients, RMSE = Root Mean Square Error, AIC = Akaike’s Information Criterion, R^2^_adj_ = the adjusted coefficient of determination, MPE = Mean Percentage Error.

### Biomass storage and compartments contributions

**Fig 3:**
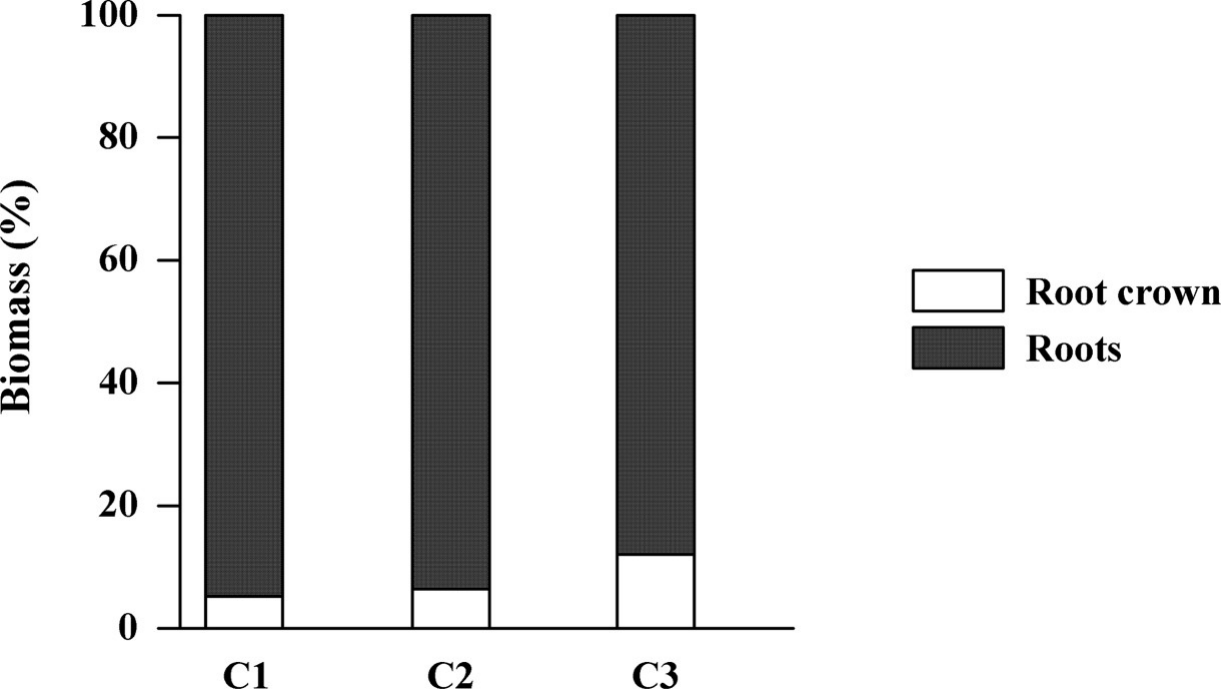
Percentage contribution of the production of the below-ground biomass per compartment. The biomass of the root crown and the roots (primary+secondary) in each height class (C1, C2, and C3) of the *Avicennia germinans* scrub forest.

The contribution of the crown to the BGB increased proportionately with increasing height class, whereas the values recorded for the roots follow the opposite pattern, that is, decreasing with increasing height (Fig 3). In height class 1, the contribution of the crown to the total biomass represents only 43% of that of class 3, whereas the contribution of the roots decline 7.4% between classes 1 and 3. A similar pattern was observed when the two compartments were analyzed together, that is, the percentage difference of the crown+roots decreased 13% between classes 1 and 3 (Fig 3).

The estimates of the mean BGB, AGB, total biomass, and the BGB:AGB ratio for each of the three scrub *A*. *germinans* height classes are shown in Table 3. Class 3, which includes the largest trees, had higher estimates of biomass, and was approximately four times more productive than class 2 and 20 times more productive than class 1. The production of biomass varied significantly among the height classes (BGB: *H* = 67.13, d.f. = 2, p < 0.001; AGB: *H* = 64.01, d.f. = 2, p < 0.001). In all cases, the *post hoc* analysis indicated that these differences were related primarily to the extremely low values recorded for class 1. No significant variation was observed when each height class was analyzed separately, however, with the production of biomass being directly proportional to the height of the vegetation.

**Table 3:** Estimated mean±standard error of the below-ground biomass (BGB), above-ground biomass (AGB), total biomass (Mg ha^-1^), and the BGB:AGB ratio, and the respective values of carbon storage recorded for each height class.

Class	BGB	BGB Carbon	AGB	AGB Carbon	Total Biomass	Total Carbon	Ratio
C1	03.26 ± 0.03Aa	01.40 ± 0.01	04.34 ± 0.06Aa	01.82 ± 0.30	07.60	03.22	0.75
C2	15.88 ± 0.30Ab	06.81 ± 0.13	17.78 ± 0.47Ab	07.45 ± 0.20	33.66	14.26	0.89
C3	64.66 ± 1.64Ac	27.74 ± 0.70	66.14 ± 2.98Ab	27.71 ± 1.28	130.80	55.45	0.98
Total	83.80	35.95	88.26	36.98	172.06	72.93	0.95

Different uppercase letters in the same line and different lowercase letters in the same column indicate significant (p < 0.05) differences between the respective values.

**Fig 4:**
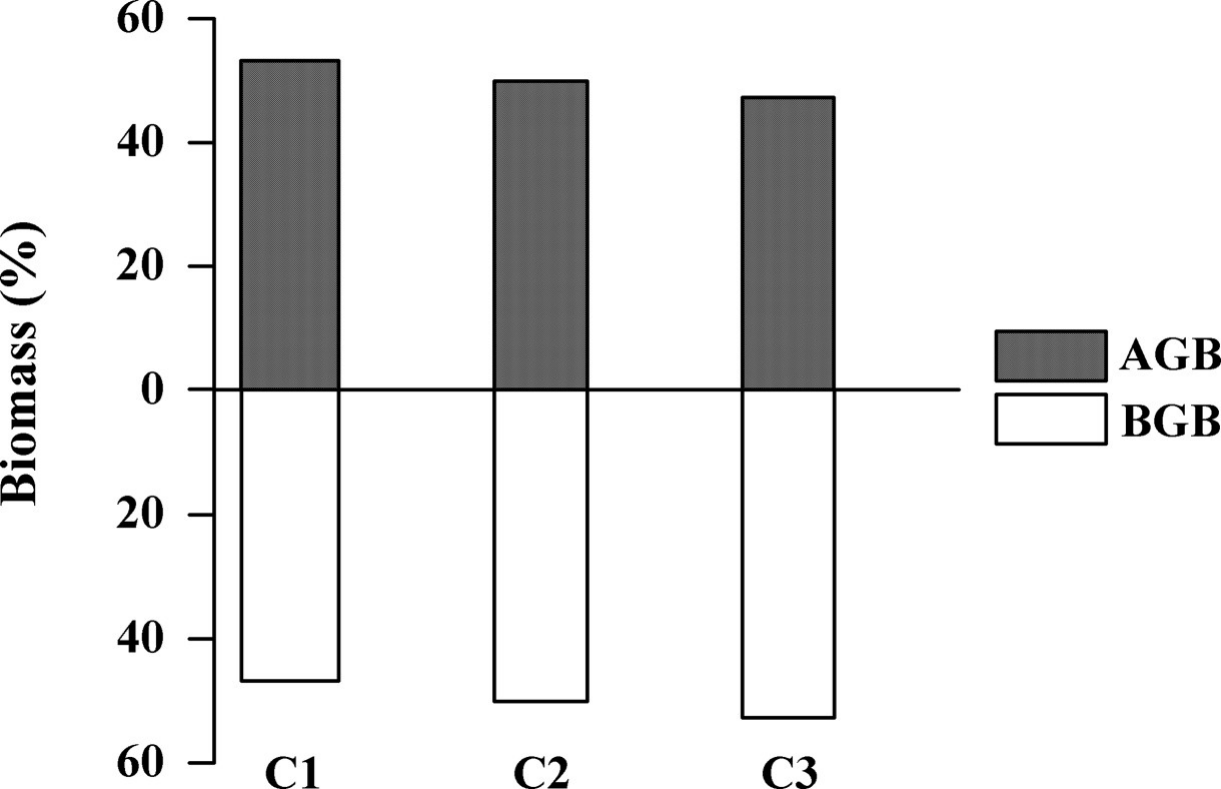
Percentage contribution of above-below ground biomass per height class. The biomass in each height class (C1, C2 e C3) of the *Avicennia germinans* scrub forest.

The results of the present study indicate an inverse relationship between the relative proportions of the BGB and AGB, and the height classes, that is, larger trees tend to produce higher BGB values that are proportionally more similar to the AGB values as a result of the increase in the percentage production of BGB and the reduction in the production of AGB (Fig 4). The estimate of the total biomass stored in the scrub *A*. *germinans* forest revealed a production of around 84 Mg.ha^-1^ of BGB (48.6% of the total biomass) and 88 Mg.ha^-1^ of AGB (51.4%). On the Ajuruteua Peninsula, the scrub mangrove forest covers an area of approximately 812 hectares, which implies a total production of approximately 139.7 Gg of biomass, and 59 Gg of carbon. Slightly more of the biomass (71.6 Gg) and the carbon (30.0 Gg) were allocated to the AGB in comparison with the BGB, with 68.0 Gg of the biomass and 28.9 Gg of the carbon (Table 4). Similarly, the biomass (kg ind^-1^) and respective C values (kg C ind^-1^) were also calculated for an average *A*. *germinans* individual of each height class (S4 Table).

**Table 4:** Estimates of the biomass (Gg) and carbon (Gg C) stocks of the dwarf *Avicennia germinans* mangrove forest by tree size classes in the root crown (RC), root (primary+secondary), and total on the Ajuruteua Peninsula in Bragança, Pará, Brazilian Amazon coast.

Class	RC	RC Carbon	Root	Root Carbon	BGB	BGB Carbon	AGB	AGB Carbon
C1	0.14	0.06	02.50	01.07	02.64	01.13	03.52	01.48
C2	0.83	0.35	12.07	05.14	12.89	05.49	14.44	06.05
C3	6.35	2.71	46.15	19.66	52.50	22.37	53.71	22.50
Total	7.32	3.12	60.72	25.87	68.04	28.99	71.67	30.03

BGB = below-ground biomass, AGB = above-ground biomass.

### Generalist allometric models *vs*. Size-specific models

A generalist model (which includes all height classes) was generated to evaluate the effects of structural differences on the estimates of the BGB of the scrub mangrove forests of the Ajuruteua Peninsula, which was y = 0.07577 * D^1.98745^ (R^2^_adj_ = 0.96; AIC = 58.69; RMSE = 0.85, and MPE = -0.77). This model underestimates by 22.6 Mg ha^-1^ (27%) the total BGB (83.8 Mg ha^-1^) derived from the sum of the estimates of the three specific equations.

## Discussion

The principal aim of the present study was to develop reliable allometric models to estimate the BGB of scrub mangrove forests, to cover an important lacuna for the understanding of the production of biomass and carbon storage in mangrove forests, given that the available models refer only to the production of AGB in this type of forest [19,27,28]. The models we developed to estimate the BGB for the different height classes found in the scrub *A*. *germinans* forest followed both linear and power trends, which is typical of the models used to estimate the biomass of tropical forests [63–69], including mangroves [21,70].

Tree height and diameter are the structural attributes included most often in our models, with no major differences in comparison with the allometric equations developed to estimate the BGB of other, non-scrub mangrove forests around the world, in either species-specific or generalist models [34,71–74]. The estimated BGB values for the different height classes of scrub mangrove forest are within the range of values reported for mangrove forests at other localities (Table 5), such as the Everglades National Park (24–47 Mg ha^-1^) and Rookery Bay (29–284 Mg ha^-1^), both in Florida, in the United States [24,25], and the Endings Lagoon (9.8 Mg ha^-1^) in Mexico [75]. Although a number of studies have provided estimates of the BGB of non-scrub mangrove forests in different parts of the world [23,76–80], data are still relatively scarce overall. Even so, broad comparisons show that our values are higher than the estimates available from the vast majority (89%) of sites in the 71 countries that have mangrove forests [81], as well as the mean value of approximately 27 Mg ha^-1^ estimated for other types of forest around the world [82]. It is important to note, however, that much of this discrepancy may be related to the effects of the application of different sampling methods, which reinforces the need for caution when comparing the results of studies based on distinct approaches [37,38,71,83].

**Table 5:** Comparison of biomass estimates (Mg ha^-1^) of the scrub mangrove forest.

	BGB Mg ha^-1^	AGB Mg ha^-1^	BGB %	AGB %
C1 (this study)	3.3	4.3	42.9	57.1
C2 (this study)	15.9	17.8	47.2	52.8
C3 (this study)	64.7	66.1	49.4	50.6
Península (this study)	83.8	88.3	48.7	51.3
Khan et al. 2007	67.0	75.1	47.1	52.9
Briggs, 1977	147.3	144.5	50.5	49.5
Briggs, 1977	160.3	141.6	53.1	46.9
Cameron, 2019	13.8	68.7	16.7	83.3
Pérez-Ceballos et al., 2017	9.8	-	-	-
Castañeda-Moya et al., 2011	24.0	-	-	-
Castañeda-Moya et al., 2011	46.7	-	-	-

BGB = below-ground biomass, AGB = above-ground biomass. C1, C2, and C3 = height classes

The BGB estimates available for mangrove forests around the world have been obtained using a range of both direct and indirect approaches, such as the trench method [26], extraction (pull up) [72], and the analysis of soil cores [79], resulting in highly diverse biomass estimates [31]. The root sampling method adopted in the present study has been applied successfully in previous studies of mangroves [38,71] and other types of tropical forest around the world [84]. The discrepancies resulting from the application of different sampling methods become especially apparent when our values for the scrub mangrove forest are compared with those obtained for other, non-scrub mangrove forest, although they present similar biomass values (Table 5). However, the estimates of BGB available for hypersaline environments are relatively low overall [85], as was the case in the present study. This reinforces the conclusion that the soil core sampling method, which focuses only on the fine roots (20 mm), will likely underestimate the BGB of mangroves [31]. Much higher values have been recorded, by contrast, in studies in which the roots are excavated, either completely (total excavation) or partially (trench, pull up, sampling method), in comparison with sampling methods that do not incorporate the roots of larger diameter [25]. Some studies have indicated that methods in which the roots are excavated provide relatively reliable estimates of the BGB, despite the fact that some of the smaller and finer parts of the root are lost during extraction [40,82].

Based on the models developed to estimate the AGB of the scrub mangrove forests of the Ajuruteua Peninsula, it was possible to estimate the total biomass of this type of mangrove. Our findings also indicate that the below-ground compartment of the scrub mangrove forests contributes a larger proportion of the biomass (48%) than that estimated for mangrove forests under minimal environmental stress, such as those studied in Tanzania, where the BGB constituted 41% of the total biomass [71]. Some studies have concluded that as much as 80% of the live forest biomass worldwide is located in the above-ground compartment, and only 20% below ground, with slightly higher percentages (~25%) being found in tropical forests [86,87]. Our results are also consistent with those of previous research which indicates that the mangroves are characterized by a relatively high percentage of BGB in comparison with other tropical forests [24,88,89]. This means that half of the total biomass and carbon stocks of the scrub mangrove forests on the Ajuruteua Peninsula is allocated to the below-ground compartment. We observed an inversely proportional relationship between the abiotic factors (topography and salinity) and the biotic variables (tree height and percentage BGB), that is, the greater the topography and the higher the salinity, the lower the height of the trees and their production of BGB, which is the exactly opposite pattern observed in the production of AGB.

However, our estimates of the production of BGB indicated a pattern that contrasted absolutely with that recorded by Saintilan [85], who found an increase in the contribution of the BGB with increasing salinity. The results of the present study also indicate higher AGB and BGB values than those recorded in a dwarf *Kandelia obovata* (S. L.) Yong forest in Japan [89]. In the present study, the percentage estimates of the biomass for height class 2 were relatively similar to those recorded in the Japanese mangrove. This is almost certainly a reflection of the structural similarities of the two types of stunted mangrove trees, given that the *A*. *germinans* trees of height class 2 were the same size as the dwarf *Kandelia* trees. However, the absolute biomass recorded in this study in Japan were similar to those of height class 3 in the present study, which may be accounted for by both the differences in the methodological approaches to the estimation of the BGB and the varying responses of the trees to the different local environmental factors.

The allocation of the biomass in the scrub mangrove forest is influenced by a range of factors, including the diameter of the tree [21,90]. Other factors, such as the local frequency of inundation, may also contribute to the dynamics of the compartmentalization of the biomass in these forests, in particular in response to environmental stressors [91–94]. This implies that fluctuations in flooding patterns also play an important role in the hydrological and/or saline stress of these environments, leading to an increase in the proportion of the BGB [23,85]. Our findings are consistent with this conclusion when the BGB estimates of the three height classes are compared. The significant variation found among classes in the BGB values may be explained by the variation in the salinity of the soil within the study site. The highest salinity was recorded in the areas dominated by shrubby *A*. *germinans* individuals from height class 1, and the lowest in areas dominated by the taller individuals from class 3. A similar tendency was found in *Avicennia marina* (Forssk.) Vierh. forests under hypersaline conditions in Australia, further reinforcing this finding [85].

Overall, our results cover a major lacuna in the models available to estimate the production of below-ground biomass and carbon storage by scrub mangrove forests, and also contribute to the refinement of the approach used to estimate total biomass in this environment. The findings of the present study indicate an inverse relationship between the stature of the vegetation and the production of BGB in the scrub mangrove forests of the Ajuruteua Peninsula, which contributes to the correction of uncertainties on the compartmentalization of the biomass and carbon in this forest. It is particularly important, in this context, to take into consideration the systematic errors in the allometric models used to estimate the BGB and AGB, given that the differences among studies can be accounted for primarily by the uncertainties intrinsic to the different models [31]. As the selection of the model is an important source of uncertainty [95], models developed specifically for each height class of the mangrove forest provide more accurate estimates, reducing the uncertainties intrinsic to the different biomass estimates (total, above- and below-ground). As major differences exist in the carbon stocks among different height classes, the specific allometric models developed for each height class should normally be applied rather than generalist models.

The results of the present study describe the effects of the gradient of topography and salinity on the production of biomass and carbon storage of scrub mangrove forests. All the models were based on direct measurements of the size and weight of the trees, which permitted the systematic calibration of the data, which reinforced the accuracy of the calculation of the tree biomass and carbon stocks of this type of mangrove forest. This implies that the site-specific models developed in the present study may be a valid option for the analysis of the extensive tract of mangrove found on the Brazilian Amazon coast, and similar coastal environments in other parts of the world, where scrub mangroves dominate much of the landscape. Ultimately, improved accuracy in the biomass estimates will be fundamental for the systematic evaluation of the process of carbon storage, and will be essential for the development of effective strategies for the conservation and management of the mangrove, as well providing potentially valuable indicators for the analysis of the impacts of climate change.

## Supporting information

S1 Table The allometric equations developed to estimate the total above-ground biomass of *Avicennia germinans* along a tree-height gradient with three classes in the scrub zone. *h* = total height (cm), D = tree diameter (cm), V = volume (cm^3^), Ca = crown area (cm^2^), a = coefficient of the response variable, b, c, d = coefficients of the predictor variables, R^2^_adj_ = adjusted regression coefficient, CF = correction factor.
(DOCX)

S2 Table Allometric models for estimating unexcavated roots biomass. (XLSX)

S3 Table Plots of standardized residuals *vs*. fitted values for regression models. (XLSX)

S4 Table Estimates of the mean±standard error of the below-ground biomass (kg ind^-1^) and carbon stock (kg C ind^-1^) of each root compartment in each height class. (DOCX)
