# Supplementary material for: Biomass and carbon estimation for scrub mangrove forests and examination of their allometric associated uncertainties
Source: PLoS One. 2020 Mar 10;15(3):e0230008. doi: 10.1371/journal.pone.0230008 (PMC7064231; doi:10.1371/journal.pone.0230008)
Supplement: S1 Table — h = total height (cm), D = tree diameter (cm), V = volume (cm3), Ca = crown area (cm2), a = coefficient of the response variable, b, c, d = coefficients of the predictor variables, R2adj = adjusted regression coefficient, CF = correction factor. (DOCX) [file pone.0230008.s001.docx]

**S1 Table**

The allometric equations developed to estimate the total above-ground biomass of *Avicennia germinans* along a tree-height gradient with three classes in the scrub zone. *h* = total height (cm), D = tree diameter (cm), V = volume (cm³), Ca = crown area (cm^2^), a = coefficient of the response variable, b, c, d = coefficients of the predictor variables, R^2^_adj_ = adjusted regression coefficient, CF = correction factor.

| Class | Predictor (x1, x2...) | *a* | *b* | *c* | *d* | R^2^_adj_ | CF |
| --- | --- | --- | --- | --- | --- | --- | --- |
| C1 | D, *h*, Ca | -3.3007 | 0.1873 | 0.7666 | 0.7003 | 0.97 | 1.00819733 |
| C2 | D, V | -3.8629 | 1.0848 | 0.5209 | n.a. | 0.99 | 1.002207767 |
| C3 | D, *h*. | -9.2154 | 1.5342 | 1.8814 | n.a. | 0.98 | 1.014860822 |

The model used was: ln(y) = a + b*ln(x1) + c*ln(x2) + d*ln(x3). Source Carneiro [16].
