# Supplementary material for: Biomass and carbon estimation for scrub mangrove forests and examination of their allometric associated uncertainties
Source: PLoS One. 2020 Mar 10;15(3):e0230008. doi: 10.1371/journal.pone.0230008 (PMC7064231; doi:10.1371/journal.pone.0230008)
Supplement: S4 Table — (DOCX) [file pone.0230008.s004.docx]

**S4 Table**

Estimates of the mean±standard error of the below-ground biomass (kg ind^-1^) and carbon stock (kg C ind^-1^) of each root compartment in each height class.

| Class | Root crown | | Root (primary+secondary) | | Total | |
| --- | --- | --- | --- | --- | --- | --- |
|  | Biomass | Carbon | Biomass | Carbon | Biomass | Carbon |
| C1 | 0.04 ± 0.004 | 0.02 ± 0.002 | 0.7 ± 0.07 | 0.30 ± 0.032 | 0.74 | 0.32 |
| C2 | 0.43 ± 0.05 | 0.18 ± 0.02 | 6.3 ± 0.68 | 2.68 ± 0.29 | 6.74 | 2.87 |
| C3 | 3.56 ± 0.52 | 1.52 ± 0.22 | 25.9 ± 3.75 | 11.03 ± 1.60 | 29.46 | 12.55 |
| Mean | 0.96 ± 0.20 | 0.41 ± 0.09 | 8.1 ± 1.42 | 3.45 ± 0.60 | 12.31 ± 8.76 | 5.25 ± 3.73 |
